# The Role of Circular RNAs in DNA Damage Response and Repair

**DOI:** 10.3390/cancers13215352

**Published:** 2021-10-26

**Authors:** Angelos Papaspyropoulos, Orsalia Hazapis, Nefeli Lagopati, Aikaterini Polyzou, Anastasios D. Papanastasiou, Michalis Liontos, Vassilis G. Gorgoulis, Athanassios Kotsinas

**Affiliations:** 1 Molecular Carcinogenesis Group, Department of Histology and Embryology, Medical School, National Kapodistrian University of Athens (NKUA), 75 Mikras Asias Str., Goudi, GR-11527 Athens, Greece; a.papaspyropoulos@med.uoa.gr (A.P.); ohazapis@med.uoa.gr (O.H.); nlagopati@med.uoa.gr (N.L.); apolizou@med.uoa.gr (A.P.); mlionto@med.uoa.gr (M.L.); 2 Biomedical Research Foundation, Academy of Athens, GR-11527 Athens, Greece; 3 Department of Biomedical Sciences, University of West Attica, GR-12462 Athens, Greece; apapanasta@uniwa.gr; 4 Histopathology Unit, Biomedical Sciences Research Center ‘Alexander Fleming’, GR-16672 Vari, Greece; 5 Oncology Unit, Department of Clinical Therapeutics, Medical School, National and Kapodistrian University of Athens, Alexandra Hospital, GR-11528 Athens, Greece; 6 Molecular and Clinical Cancer Sciences, Manchester Cancer Research Centre, Manchester Academic Health Sciences Centre, University of Manchester, Manchester M20 4GJ, UK; 7 Center for New Biotechnologies and Precision Medicine, Medical School, National and Kapodistrian University of Athens, GR-11527 Athens, Greece; 8 Faculty of Health and Medical Sciences, University of Surrey, Surrey GU2 7YH, UK

**Keywords:** circRNA, double-strand breaks (DSB), DNA damage response and repair (DDRR), tumorigenesis

## Abstract

**Simple Summary:**

The role of non-coding RNA, and particularly of circular RNA, in the DNA damage response and repair network is underappreciated. Given the vital role of this network in preserving the genomic integrity and consequently cellular homeostasis, the constantly increasing numbers of discovered circular RNAs and the increasing implication of these molecules in the function of this network unravel a new important field that may open new therapeutic opportunities, but also require detailed investigation.

**Abstract:**

Circular RNAs (circRNA) comprise a distinct class of non-coding RNAs that are abundantly expressed in the cell. CircRNAs have the capacity to regulate gene expression by interacting with regulatory proteins and/or other classes of RNAs. While a vast number of circRNAs have been discovered, the majority still remains poorly characterized. Particularly, there is no detailed information on the identity and functional role of circRNAs that are transcribed from genes encoding components of the DNA damage response and repair (DDRR) network. In this article, we not only review the available published information on DDRR-related circRNAs, but also conduct a bioinformatic analysis on data obtained from public repositories to uncover deposited, yet uncharacterized circRNAs derived from components of the DDRR network. Finally, we interrogate for potential targets that are regulated by this class of molecules and look into potential functional implications.

## 1. Introduction

Chromosomal rearrangements following incorrect repair of DNA double-strand breaks (DSB) constitute one of the primary causes of tumorigenesis, setting the grounds for genomic instability [1]. Several factors can lead to DNA damage, including ionizing and UV radiation, oncogene activation, exposure to chemical carcinogens and viral infections [1,2]. DSB formation is more frequently observed in the proximity of DNA:RNA hybrids known as R-loops [3,4]. R-loops can form in the process of transcription or during the interaction of DNA with regulatory RNAs resulting in DNA double strand separation, which renders DNA more vulnerable to genotoxic stress [5,6]. To safeguard the integrity of the genome, cells have developed DNA damage response and repair (DDRR) pathways, whose role is to respond to genotoxic insults [1,2]. DDRR circuits comprise the subject of intense ongoing research, as alteration of their function results in accumulation of genomic instability leading to oncogenic transformation [1,2]. 

**Figure 1:**
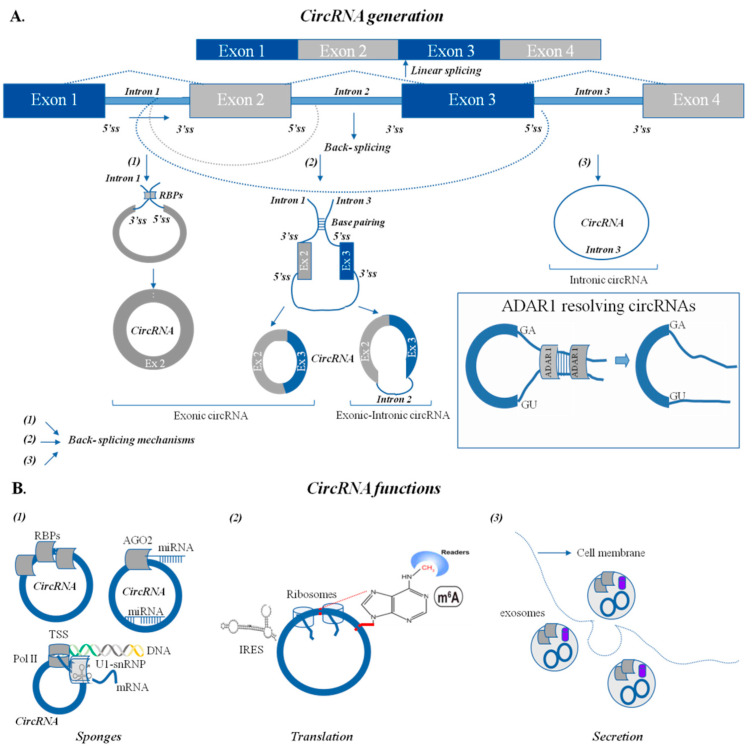
Mechanisms that lead to back-splicing events and circRNA functions. (**A**). (1) The first mechanism involves RNA-binding proteins (RBPs); binding to the flanking intron creates a closed loop that brings the complementary splice donor and acceptor sites close. As a result, an exoniccircRNA is generated. (Ex: exon) (2) The second form of back-splicing has to do with complementary introns within ALU repeats that bring the exons closer, resulting in the formation of either exonic circRNAs initiated by the 3′ end exon splice donor site joined to the 5′ end of an upstream exon or the 3′ end exon splice donor site joined to the 5′ end of an upstream exon with a retained intron. The latter can also be formed via the binding of RBPs at the flaking intron position between exon 2 and 3. Consequently, exonic circRNAs or exonic-intronic circRNAs are generated. (3) The third type of back-splicing results only from intron pairing, where splicing is prompted by reverse complementary sequences within ALU repeats (located in the upstream and downstream introns). This process results in intronic circRNA production. **Inset:** The circular RNA formation can be degraded via ADAR1, whose binding at double stranded regions, such in the case of base-pairing introns within ALU repeats, can break-down the circular formation [27]. (**B**). CircRNAs function as: (1) sponges that bind proteins or other nucleic acids, (2) can be translated, and (3) are secreted via exosomes.

An increasing body of evidence has demonstrated that several types of RNA species hold critical roles in various nuclear processes, such as DNA replication and repair, chromosome structure regulation, telomere elongation and chromatin organization [4,7,8,9]. A large number of eukaryotic protein-coding genes have been found capable of generating exonic circular RNAs (circRNAs), which may exist at higher levels than their respective linear mRNAs [10], as the circRNAs have an increased life due to resistance to RNA degradation via exonucleases and can thus accumulate to levels that can even exceed the life of their cognate linear mRNAs [11]. CircRNAs are generally classified as non-coding RNAs (ncRNAs), which unlike linear RNA, are covalently closed RNA loops acting as mammalian gene regulators (Figure 1) [12,13]. Nevertheless, recent data have challenged this view by demonstrating that certain circRNAs can support translation to produce functional peptides [14]. Although circRNAs were originally regarded as splicing errors of low abundance, they have been recently shown to be highly abundant and evolutionarily conserved in eukaryotes, where they are expressed in a tissue-specific fashion [12,13,15,16]. CircRNAs are produced by exons or lariat introns by a process called back-splicing, whereby the 3′ and 5′ ends normally encountered in an RNA molecule are covalently joined together in a circular structure (Figure 1) [12,13]. Through their cis and trans functions, circRNAs have been found to regulate important oncogenes and tumor suppressors, including major players of the DDRR network [10]. Several methodologies have been reported for the identification of circRNAs, demonstrating that their aberrant expression pertains to a variety of pathological conditions, including cancer [10].

Herein, we focus on the various mechanisms through which circRNAs regulate the function of important genes of the DDRR network, thus exerting a direct impact on genomic stability and cancer progression. Moreover, we conducted a bioinformatic analysis to reveal potential circRNA isoforms that derive from the linear RNAs of key components of the DDRR network and explore their possible role in cell fate regulation.

## 2. Biogenesis, Function and Role of circRNAs in Cancer

CircRNAs, together with microRNAs (miRNAs) and other ncRNAs, comprise about 95% of total RNA in eukaryotes, and an emerging body of evidence suggests their active involvement in gene regulation [12]. CircRNAs derive from the back-splicing of exons, introns or both, leading to their classification as exonic, intronic and exonic-intronic circRNAs, respectively (Figure 1) [15]. CircRNA transcripts were first discovered over three decades ago, but their role was undermined as they were originally thought to represent RNA splicing errors [12]. The identification of circRNAs in cancerous and non-cancerous cell line models and Acute Lymphoblastic Leukemia (ALL) patients [17] led to an increasing interest in circRNA biology. Recent research in the circRNA field has, thus, resulted in the discovery of a large number of circRNAs which are considerably more stable and abundant than their linear counterparts in mammalian cells [18].

Exonic circRNAs may be generated from single or multiple exons, and are the product of pre-mRNA splicing, where a 3′ splice donor is attached to a 5′ splice acceptor yielding a circular structure (Figure 1) [15,18]. In cases where the intron between exons is maintained, the derivative structure is referred to as exonic-intronic circRNA, while intronic circRNAs are generated from intron lariats which have not been degraded by de-branching enzymes (Figure 1A) [12,19]. In metazoans, it has been found that the process of back-splicing is likely carried out by the spliceosome [13,20,21,22,23]. Inhibition of the spliceosome via the pre-mRNA splicing inhibitor isoginkgetin decreases circRNA levels, indicating that the spliceosome may indeed hold an important role in circRNA generation [22]. As circRNA levels do not always correlate with the levels of the respective linear transcripts, it has been inferred that circRNA expression is under strict control, while the spliceosome is able to discriminate between canonical linear splicing and back-splicing [24]. Particularly, the frequency of back-splicing events compared to canonical splicing has been reported to be low and less efficient [25]. Moreover, it has been shown that approximately only 20% of the protein coding genes in the brain produce circRNAs [25]. Nevertheless, it has been demonstrated that several hundreds of circRNAs in the brain are expressed more than their canonical linear isoform [26]. In addition, different regions of the brain were shown to have different types of circRNAs increased. 

Of note, highly expressed circRNAs have been shown to be produced from exons, particularly from those located closer to the transcription start of the host gene [26]. Moreover, as demonstrated, the introns flanking conserved circRNAs often contain reverse complementary matches (RCMs), and in the brain, these introns are often more than 10 kb in length. Quite interestingly, introns in the 5′UTRs are, on average, longer than introns in the CDSs and 3′UTRs [28], which could reflect that more RCMs or regulatory sequence domains recruit different spliceosomal machinery leading to the production of back-splicing events. Furthermore, circRNAs are resistant to exonuclease digestion, which results in an RNA half-life of ~18 h–27 h [11], which makes them by far a very stable RNA molecule as the average half-life of an RNA is ~4 h–7 h.

A recent body of evidence has suggested that circRNAs may be implicated in the initiation and progression of tumorigenesis [29,30]. Over 27,000 circRNAs were recently collectively identified in non-cancerous and cancerous human tissues [31]. Importantly, circRNAs have been found to be downregulated in some tumor lesions in comparison to healthy tissue, which has been attributed to the presence of back-splice machinery errors in tumor lesions, miRNA-mediated degradation of circRNAs and reduction due to accelerated cellular proliferation in tumor cells [12,32]. In support of that, it has been shown that circRNAs are more likely downregulated in colorectal cancer cell lines carrying mutant KRAS, in comparison to wild-type counterparts [33]. 

The role of circRNAs in cancer has been the object of recent research efforts showing a relationship between circRNAs produced by established oncogenes and oncogenic outcome. For example, cir-ITCH, which functions as an miRNA sponge to increase ITCH expression levels, is found downregulated in colorectal cancer tissues compared to control [34]. This is in line with an identified role for ITCH as an inhibitor of the Wnt signaling pathway which holds a prominent role in colorectal cancer development and progression [34]. Along the same lines, circ-BMI1 was also recently found deregulated in esophageal cancer [35]. BMI1 is an important Notch signaling target, involved in neuroprotection [36,37]. Circ-BMI1 induction resulted in reduced proliferation and migration of tumor cells, implying its potential implementation in esophageal cancer diagnosis and treatment [35]. Moreover, treatment of LNCaP prostate cancer cells with the proliferation inhibitor dinaciclib resulted in a marked increase of circRNA levels regardless of changes in the expression of parent genes, corroborating the notion that circRNAs may be reduced by cell division [10,38]. 

Interestingly, it has been suggested that circRNAs may control the biological activity of a network of competing endogenous RNAs (ceRNAs) [39]. According to the ceRNA hypothesis, a variety of RNA species regulate genomic expression post-transcriptionally, implying that mRNAs, pseudogene transcripts, lncRNAs and circRNAs may affect the half-life or translation of target RNAs via competition for binding to the same miRNA [39]. It has been shown that circRNAs act as ceRNAs to regulate GDNF family receptor alpha-1 (GFRA1) expression via modulating miR-34a levels, thus exerting anti-apoptotic functions in triple-negative breast cancer [40]. An emerging body of evidence suggests that circRNAs may act as ceRNAs to regulate important biological properties related to tumorigenesis, such as proliferation, angiogenesis and apoptosis [39]. 

It is, thus, becoming clear that circRNAs are associated with cancer patient clinical outcomes, by exerting important functions in cancer cells [41,42,43]. CircRNAs may also have opposite roles from their linear counterparts, as is the case with a circRNA encoded by the mouse and human *Zbtb7a* gene which has a proto-oncogenic role in mesenchymal tumors, while the respective linear RNA acts as a tumor suppressor [44]. Other circRNAs, such as the one derived from the mouse or human *Foxo3* gene, induce apoptosis, thereby restricting tumor growth [45,46]. Additionally, as circRNAs have been identified in exosomes and body fluids, they hold great promise as novel disease biomarkers [43,47,48]. 

## 3. Capturing circRNA-Protein and -miRNA Interactions

To enrich for circRNAs, ribonucleases can be used to remove rRNA, tRNA, poly(A)+ RNAs, and then preserve the circular forms with RNAse R treatment, which will degrade only the linear RNAs. To identify a putative site for RBP binding, RPA assays can be used using RNAse-H, which will digest the unprotected unbound-RNA site [49]. To investigate putative circRNA-interactions, DNA oligo probes conjugated with streptavidin-coated magnetic beads can also be used to capture and pull-down the circRNA and the proteins bound as demonstrated in [45]. Another strategy that can be employed is the use of glycerol gradient centrifugation to capture circRNA-protein complexes of distinct sizes which can be followed by RT-PCR to determine abundant circRNAs [50]. Fluorescently labeled antibodies can be used to target and isolate protein-circRNA complexes. Furthermore, RNA pull-down assays with luciferase reporters can be used [51], to investigate potential RNA-RNA interactions between circRNA, miRNA and mRNAs. 

Experimental evidence of translation has been previously shown [52], where Circ-ZNF609 was found to be associated with heavy polysomes, in a cap-independent manner. Subsequently, the authors generated an expression vector that was able to produce circular transcripts [53]. A construct containing a 3xFLAG coding sequence upsteam of a stop codon was produced only upon formation of a circular RNA, which led the authors to demonstrate that circ-ZNF609 can be translated. In another report [54], the authors demonstrated that the m^6^A-driven translation of circRNAs along with IRES elements is widespread. To achieve this, a minigene reporter containing split GFP and a viral IRES that could be efficiently translated was generated.

## 4. Towards a Unified Nomenclature for Circular RNAs

As new biochemical/NGS protocols are emerging together with novel bioinformatic approaches, more evidence will be provided towards the distinction and classification of circRNAs, thus posing the need for a standard nomenclature, as circRNAs become essential biomarkers of disease. The current nomenclature derives from circBase, which includes the species and a numeric code, while circBank and circAtlas use the gene symbol of the transcript that results in the generation of a circRNA annotation based on the genomic coordinates from UCSC (https://genome.ucsc.edu/, accessed on 8 September 2021). CIRCpedia uses a different nomenclature which includes the species and a number derived internally as an identifier for circBase. Furthermore, as previously demonstrated [55], there has been a recent effort to provide a unique nomenclature, likewise with miRNAs, such that the species are represented by the first letters, followed by the gene name and the exons involving the circularization: eg hsa-circ-gene_name-(exons7-8).

## 5. DNA Damage Response and Repair (DDRR) Pathways

Genomic integrity is crucial not only for cellular growth, but also for transmission of intact genetic information to daughter cells upon cellular division [56]. However, there are various types of internal and external genotoxic insults which challenge the integrity of the genome, leading to the activation of compensatory signaling pathways in order to rectify DNA damage and restrict genomic instability [1,2]. DNA damage sensors constitute the first DDRR pathway components to identify DNA damage sites and subsequently activate signal transduction routes depending on the type of damage, by directly recruiting DDRR proteins at those sites [56,57]. DNA damage sensors frequently coexist with signal transduction molecules; therefore, their distinction is often difficult [56]. 

Two main types of genetic aberrations are encountered in the genome: changes at the nucleotide level and single/double strand breaks (SSBs/DSBs) [1,58]. DSB formation is the most deleterious type of damage as DSBs become lethal upon insufficient repair, whereas, if they are incorrectly repaired, they constitute a source of genomic instability setting the grounds for disease, including tumorigenesis [1,58]. Among the consequences of defective DSB repair is the occurrence of genomic rearrangements, with a considerable impact on the genome integrity, often culminating in oncogene activation [1,58,59]. 

Upon DSB formation, two primary repair pathways are triggered, the Non-Homologous End Joining (NHEJ) and the Homologous Recombination (HR) pathway [1,60,61]. One of the most important DNA damage sensors in the DDRR process is H2AX, a variant of the H2A histone, which upon DSB formation is phosphorylated and forms γH2AX loci [62]. The NHEJ pathway is activated through the Ku70/Ku80 heterodimer (simply referred to as Ku), which selectively identifies DSBs [63]. Clinically, it was shown that, in B cell chronic lymphocytic leukemia patients, a subset of B cells were resistant to radiation-mediated apoptosis, which was accompanied by an increased DNA binding ability of Ku [64]. Briefly, binding of the Ku70/Ku80 heterodimers to a DSB leads to the formation of the DNA-PK complex, which together with the Artemis nuclease are implicated in processing DNA ends [1]. Finally, the XLF-XRCC4-DNA ligase IV complex completes the NHEJ process, which is by nature an error-prone mechanism as the DNA ends are joined directly [1]. 

The HR pathway occurs during the S and G2/M phases of the cell cycle and engages the MRN complex (MRE11-RAD50-NSB1), which functions as an intermediate link between DSB formation and cell cycle checkpoint activation [65]. It was shown that the MRN complex acts as a sensor of DNA damage and facilitates the recruitment of Ataxia-Telangiectasia Mutated (ATM) to DNA damage sites [66]. Following DSB recognition, DNA end resection is carried out by CtIP and EXO I, resulting in RPA coating of the derivative single-stranded 3′ overhangs, which enables RAD51 loading [1]. The RAD51 nucleoprotein filament is then extended along the homologous chromatid DNA by DNA polymerase [1]. Given that sister chromatid DNA is used as the repair template, the HR pathway is generally regarded as an error-free process. 

BRCA1 C-Terminal (BRCT) domains are important modules mediating protein–protein interactions in DDRR pathways [56]. BRCT domains have been identified in important DDRR network components, including NBS1, 53BP1 and BRCA1 [56]. 53BP1 is an important player in DDRR, whose H4K20me2/H2AK15ub-mediated recruitment to DNA damage sites results in 53BP1-H2AX-pS139 binding, which is required for pATM accumulation at the site of damage [67]. 

The hereditary breast and ovarian cancer biomarkers BRCA1 and BRCA2 are also pivotal in DSB repair in the HR pathway [68]. Following radiation exposure, BRCA1 forms a complex with RAP80-Abraxas to associate with ubiquitinated histones [69]. Additionally, BRCA1 can interact with the SWI2 family member CSB as well as the MRN complex to facilitate DNA end resection [70,71]. Because of their so far identified functions, BRCA1/2 have become valuable biomarkers in predicting radiotherapy outcomes [56].

## 6. circRNAs and DDRR

### 6.1. circRNAs as Regulators of DDRR Network Components and Genotoxic Stress in Cancer

The expression profile of circRNAs in colorectal cancer tissues compared to their normal counterparts showed that hsa-circ-101555 was markedly elevated in tumor samples, correlating with patient prognosis [72]. Hsa-circ-101555 was produced via back-splicing of the *CSNK1G1* gene and demonstrated higher stability than the respective linear RNA [72]. Interestingly, Hsa-circ-101555 silencing was found to suppress proliferation, activate apoptosis, and impair the DDRR in vitro and in vivo [72]. Mechanistically, hsa-circ-101555 was identified as a “sponge” of miR-597-5p whose target is the cell cycle regulator CDK6, indicating that hsa-circ-101555 may act as a competitor of miR-595-5p in upregulating CDK6 expression in colorectal cancer [72]. 

The involvement of circRNAs in the p53 pathway was examined in the HCT116, RKO and SW48 colorectal cancer lines, which were either left untreated or treated with a DNA-damaging agent [73]. Interestingly, in contrast to the high amount of mRNAs produced upon DNA damage in response to p53 activation, only a few circRNAs were found upregulated such as circ-MDM2 (hsa_circ_001371), which is produced from the *MDM2* gene [73]. MDM2 is a p53 transcriptional target, also negatively regulating p53 stability and activity [74,75]. Circ-MDM2 (hsa_circ_001371) knockdown resulted in p53 upregulation and was accompanied by growth defects in vivo and in vitro [73]. In line with those results, several p53 targets were found increased, while retinoblastoma (Rb) phosphorylation was reduced and G1/S transition was deregulated upon circ-MDM2 loss, implying that circ-MDM2 may be a p53 and cell cycle progression regulator [73]. 

In a study investigating the link between circRNAs and tumor grade in bladder cancer patients, a fraction of circRNAs (157 of a total of 4571 circRNAs) were differentially expressed among various tumor grades compared to the respective linear transcripts [76]. The study implied that circRNAs with target sites for miR-204-5p/211-5p may affect DDRR pathways, as the duo of miR-204-5p/211-5p targets many genes, including the DNA damage response players CDC42 and MDM2 [76].

Another study demonstrated that, contrary to the *SMARCA5* gene, circ-SMARCA5 (hsa_circ_0001445) is downregulated in clinical breast cancer samples compared to normal control tissues [77]. SMARCA5 is an established chromatin remodeler, with an active role in DDRR as it is rapidly activated in response to DSBs [78,79]. Ectopic expression of circ-SMARCA5 (hsa_circ_0001445) was sufficient to render breast cancer cell lines sensitive to drugs, both in vitro and in vivo. Additionally, it was shown that circ-SMARCA5 forms an R-loop from binding to its parental gene locus, leading to transcriptional pausing [77]. The study concluded that circ-SMARCA5 increases sensitivity to cytotoxic drugs, as its expression caused SMARCA5 downregulation [77]. 

RecQ-mediated genome stability protein 1 (RMI1) plays an important role in genome stability maintenance as part of the BLM-Topo IIIa-RMI1-RMI2 complex [80]. RMI1 knockdown was found to increase radiosensitivity and apoptosis [81]. RNA sequencing analyses in human embryonic kidney 293T cells demonstrated a number of differentially expressed circRNAs upon RMI1 knockdown, which were implicated in histone H3K36 methylation and the mismatch repair pathway, among other biological processes [81]. Those data indicated that, apart from the critical role of RMI1 in inducing ionizing radiation (IR) sensitivity, circRNAs exert a regulatory function over the IR response process [81]. Along the same lines, He et al. attempted to investigate the differential circRNA expression between irradiated and non-irradiated HEK 293 cells, and found downregulation of hsa_circ_0000734 post irradiation leading to RNF168 reduction [82]. As low RNF168 levels impair the DDRR process resulting in increased tumor incidence [83], the study provided additional insight into the role of circRNAs in IR response.

A recent study in glioblastoma cells showed that low-dose radiation triggered the secretion of exosomes containing high levels of circ-METRN, which in turn led to increased γH2AX [84]. This suggested the presence of an efficient DDRR process in glioblastoma cells. Circ-METRN was found to be responsible for tumor progression and resistance to treatment, hence exhibiting an oncogenic function through deregulation of the DDRR [84]. Another study on exosomes investigated the biology of exosomal circ-DB (circ-deubiquitination, hsa_circ_0025129), which is reciprocally related to miR-34 and negatively associated with the DNA repair player USP7 in hepatocellular carcinoma patients [85]. The study showed that adipose-derived exosomes may function as circ-DB carriers, promoting cancer growth and inhibiting DNA damage by binding miR-34a and triggering the USP7/CyclinA2 pathway in vivo and in vitro [85].

A number of studies have shed light on the role of miRNAs, lncRNAs and circRNAs in regulating genotoxic responses triggered by various environmental or internal stimuli. An interactive network among circRNA, lncRNA and miRNA has been identified in response to lead-induced neurotoxicity [86,87]. MiR-671, which is a target of lncRpa and circ-Rar1, is a negative apoptosis regulator as it targets apoptosis-related mRNAs, such as Akt2 and caspase 8 [87]. Caspase 8 is inhibited by Plasminogen Activator Inhibitor-1 (PAI-1) in cancer [88,89,90], implying a potentially clinically significant connection between miR-671/circ-Rar1 and PAI-1. In a mouse model of lead-induced neurotoxicity, lncRpa and circ-Rar1 levels are increased in the hippocampus downregulating miR-671 expression, which leads to apoptosis [87]. Moreover, miR-671 is part of a feedback loop negatively regulating circ-Rar1 [87]. Although those findings mostly pertain to neurotoxicity, the identified mechanism could be extrapolated to tumorigenesis as it is activated in response to DNA damaging genotoxic insults.

**Figure 2:**
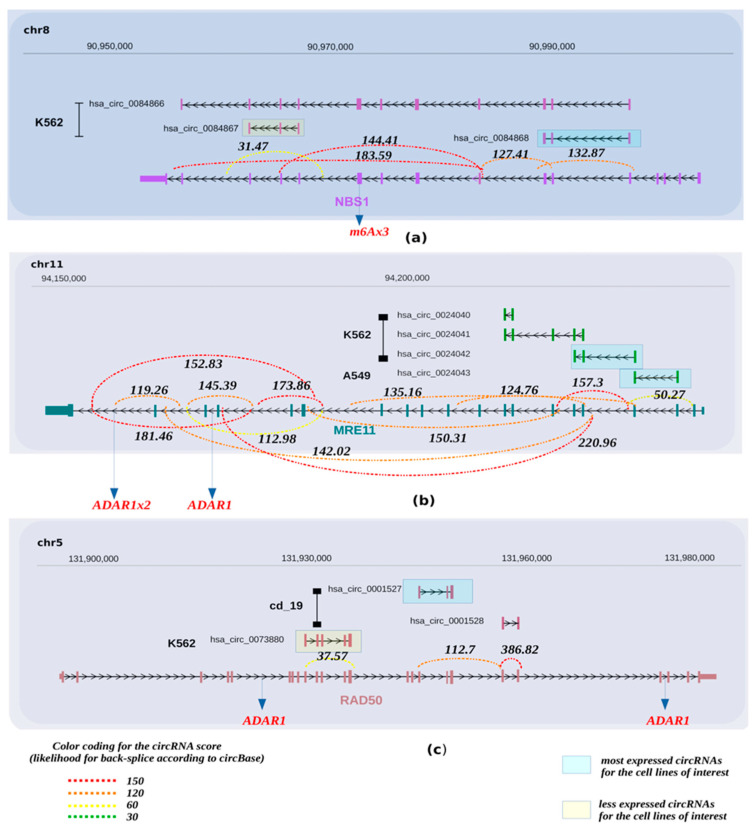
Potential circRNAs derived from the *NBS1*, *MRE11*, *RAD50* gene loci. Predicted circRNAs at the *NBS1* (**a**), *MRE11* (**b**), *RAD50* (**c**) gene loci, based on next generation sequencing (NGS) data, as deposited at circBase and CIRCpedia, are shown over the corresponding loci, based on human genome GRCh37/hg19. Cell lines in which NGS was performed to obtain the depicted circRNAs are also shown and were retrieved from circBase. The expression values for the estimated circRNAs were derived from the junction reads form of circBase and CIRCpedia from Ribo zero RNA-seq. The strength of the back-splicing event is demonstrated using a dashed line, where differentscores are shown for the expressed back-spliced events. Furthermore, we indicate from miCLiP experiments [91] a consensus of m6A sites and editing sites as derived from RNA editing events using the Jacussa pipeline [92] as well as from ADAR1 CliP binding sites.

### 6.2. Identification of Novel DDRR-Derived circRNAs from Bioinformatics Analyses

Currently and to the best of our knowledge, it is unknown if circRNAs are generated during the transcription of components of the DDRR network. Moreover, if such DDRR-derived circRNAs are produced, important unmet issues are if and how they affect the expression of the genes they originate from and/or of other genes as well as their overall impact on a cell’s fate. 

**Figure 3:**
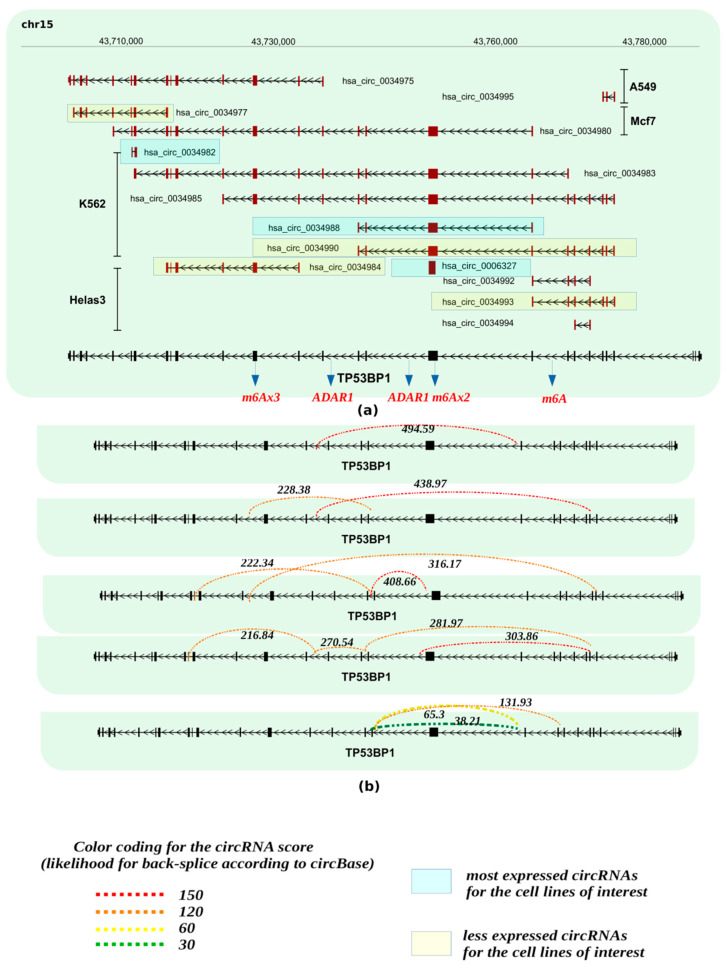
Potential circRNAs derived from the *TB53BP1* gene locus. (**a**) Predicted circRNAs at the *TB53BP1*gene locus, based on NGS data, as deposited at circBase and CIRCpedia are shown over the corresponding loci, based on human genome GRCh37/hg19. Cell lines in which NGS was performed to obtain the depicted circRNAs are also shown and were retrieved from circBase. (**b**) The expression values for the estimated circRNAs were derived from the junction reads, the form of circBase and CIRCpedia from Ribo zero RNA-seq. The strength of the back-splicing event is demonstrated using a dashed line, where different scores are shown for the expressed back-spliced events. Furthermore, we indicate from miCLiP experiments [91] a consensus of m6A sites and editing sites as derived from RNA editing events using the Jacussa pipeline [92] as well as from ADAR1 CliP binding sites.

**Figure 4:**
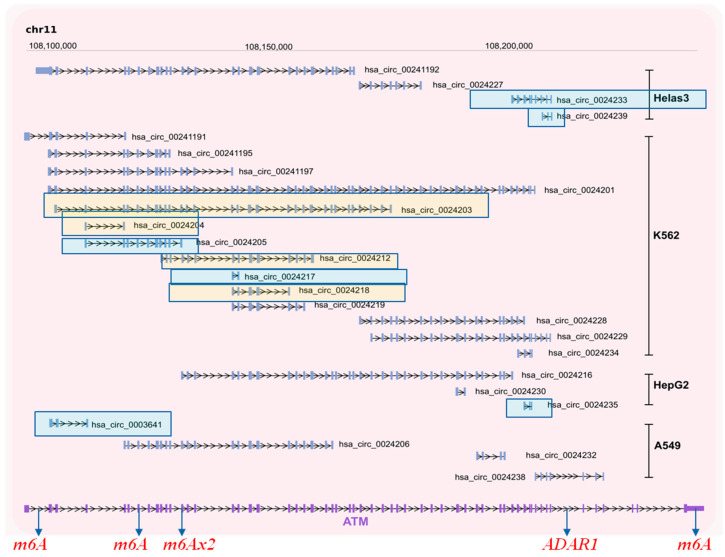
Potential circRNAs derived from the *ATM* gene locus. Predicted circRNAs at the *ATM*gene locus, based on NGS data, as deposited at circBase and CIRCpedia are shown over the corresponding loci, based on human genome GRCh37/hg19. Cell lines in which NGS was performed to obtain the depicted circRNAs are also shown and were retrieved from circBase. For more details, see Supplementary Figures. Furthermore, we indicate from miCLiP experiments [91] a consensus of m6A sites and editing sites as derived from RNA editing events using the Jacussa pipeline [92] as well as from ADAR1 CliP binding sites.

**Figure 5:**
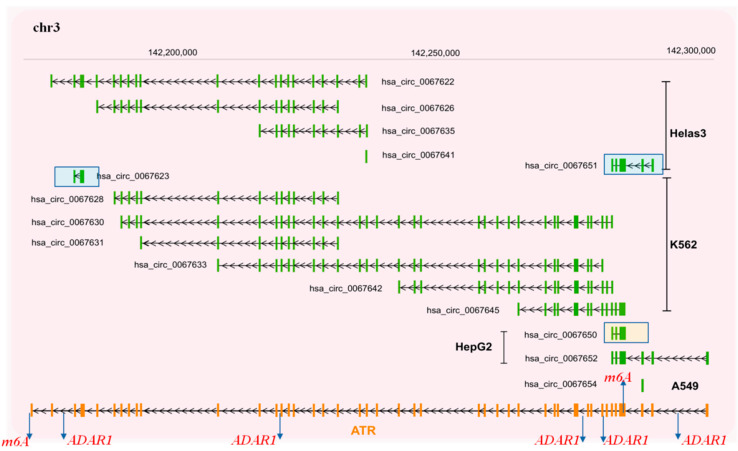
Potential circRNAs derived from the *ATR* gene locus. Predicted circRNAs at the *ATR*gene locus, based on NGS data, as deposited at circBase and CIRCpedia are shown over the corresponding loci, based on human genome GRCh37/hg19. Cell lines in which NGS was performed to obtain the depicted circRNAs are also shown and were retrieved from circBase. For more details, see Supplementary Figures. Furthermore, we indicate from miCLiP experiments [91] a consensus of m6A sites and editing sites as derived from RNA editing events using the Jacussa pipeline [92], as well as from ADAR1 CliP binding sites.

**Figure 6:**
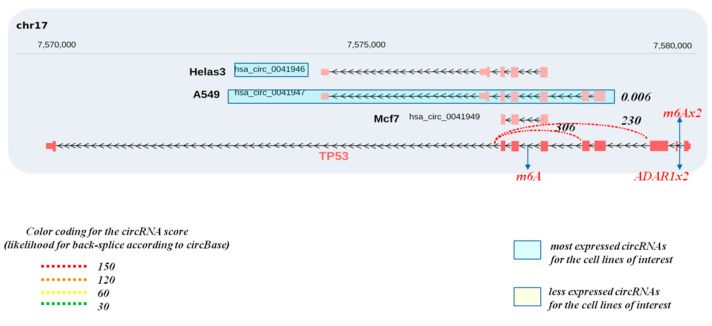
Potential circRNAs derived from the *TP53* gene locus. Predicted circRNAs at the *TP53*gene locus, based on NGS data, as deposited at circBase and CIRCpedia, are shown over the corresponding loci, based on human genome GRCh37/hg19. Cell lines in which NGS was performed to obtain the depicted circRNAs are also shown and were retrieved from circBase. The expression values for the estimated circRNAs were derived from the junction reads form of circBase and CIRCpedia from Ribo zero RNA-seq. The strength of the back-splicing event is demonstrated using a dashed line, where different scores are shown for the expressed back-spliced events. Furthermore, we indicate from miCLiP experiments [91] a consensus of m6A sites and editing sites as derived from RNA editing events using the Jacussa pipeline [92] as well as from ADAR1 CliP binding sites.

To identify whether circRNAs can be produced from gene loci encoding DDRR components, we interrogated the circBase database (http://www.circbase.org/, accessed on 8 September 2021). This database encompasses public circRNA datasets obtained from NGS of transcriptome from various tissues and mainly cell lines, which were processed for the potential presence of circular RNAs derived from back-splicing events from the genes of interest. Given that the DDRR network is vast, consisting of many components [2], we focused mainly on the following core components; NBS1, MRE11 and RAD50 that form the MRN adaptor complex; TP53BP1, a factor involved in tipping the balance between HR and NHEJ DNA repair routes; the apical ATM, ATR and downstream CHK1/CHK2 kinases, and finally TP53, a key downstream DDRR effector [2] (Figure 2, Figure 3, Figure 4, Figure 5 and Figure 6 and Supplementary Figure S1). Several isoforms were found for each genomic locus depending on the cell line, while the scores that identify the most reliable back-splicing events are depicted in Supplementary Figures S1–S9. 

Of note, the information deposited in circBase for these circRNAs has been obtained mostly from human cancer cell lines. This raises the question of whether DDRR-derived circRNAs produced in a disease-free physiological context, like in normal tissues and cells, may differ qualitatively and/or quantitatively. In other words, are the same and/or isoforms expressed in all normal cells and at what levels?

### 6.3. Potential Targets and Products of the DDRR-Derived circRNAs

Given that circRNAs can interact and “sponge” other molecules, like proteins and miRNAs, we next proceeded to identify all potential RNA binding proteins (RBPs) and miRNA binding sites that are present within the sequences of the circRNAs originating from the above DDRR hallmark genes (https://circinteractome.nia.nih.gov/, https://dorina.mdc-berlin.de/, accessed on 8 September 2021) [93] (Supplementary Figures S1–S9, Supplementary Table S1). Notably, all RBPs and miRNAs found to be potential binding targets for the circRNAs of the above DDRR genes were bioinformatically examined for the possibility to cluster within common gene ontology functions. While no clear signature was found across all RBPs, at a single circRNA level, a wide spectrum of such factors were found with potential to bind and with varying frequency (Supplementary Figures S1–S9, Supplementary Table S1B). Nevertheless, experimental verification is needed to confirm which of these interactions occur in vivo. On the other hand, the analysis of the miRNAs regarding their potential targets and end cellular effect revealed that many of them are involved in network(s) related to cancer development (Supplementary Table S1D). This finding, although not experimentally validated, raises an intriguing question; given that the DDRR network through its protein components safeguards the genome integrity from the deleterious effects of DNA double strand breaks and other genotoxic insults, what is the impact from these circRNAs on the regulation of cellular homeostasis and particularly malignant transformation? Specifically, it would be interesting to define whether these circRNAs add or not to the antitumor activity exerted by the protein counterparts encoded by the DDRR genes [1], through their “sponging” activity towards oncogenic factors and/or possibly DDRR suppressors in addition to if and how these circRNAs are deregulated in various malignancies and what would be the impact in such cases. To address this issue, we retrieved single nucleotide mutational profiles (SNPs) from various sources, such as the Circvar (http://soft.bioinfo-minzhao.org/circvar/, accessed on 8 September 2021) and the Cosmic (https://cancer.sanger.ac.uk/cosmic, accessed on 8 September 2021) databases that have been reported in various malignancies, and examined their overlap with the circular RNA transcripts derived from the investigated DDRR coding genes. Interestingly, we noted a significant overlap of reported SNPs within the sequences of the DDRR-related circRNA (Supplementary Figures S1–S9C), suggesting that their binding ability is altered in tumors, signifying a potential deregulation of their function (Figure 1B). Of note, in the current manuscript, we explored bioinformatically only a small fraction of components of the DDRR network. Interrogating a wider spectrum of DDRR genes for circRNA expression, their corresponding functions and alterations that accumulate will provide a more thorough picture on the role of the DDRR and hopefully provide targets for treatment and/or prediction options. 

Finally, to investigate the potential presence of Internal Ribosome Entry Site (IRES) elements, and therefore the possibility that the generated circRNAs are translated, we first used the circRNAfasta files from circBase, next extracted the secondary RNA structure with Vienna (http://rna.tbi.univie.ac.at/, accessed on 8 September 2021) and finally searched for candidate IRES elements that can promote translation using the IRESite tool (http://iresite.org/IRESite_web.php?page=search, accessed on 8 September 2021) (Supplementary Figure S1 and Supplementary Table S1). Although IRES elements could not be found for these core components, this warrants experimental validation, employing methods described in Section 3.

## 7. Implementation of New Models in circRNAResearch against Cancer

To investigate the role of circRNAs in cellular (patho)physiology, 2D and 3D cellular systems are being employed as adequate investigation platforms. In the case of 2D cellular systems, inducible systems overexpressing DDRR-related genes, like the human bronchial epithelial cells with Tet-ON inducible expression of the CDC6 replication licensing factor (HBEC-CDC6 TET-ON) [94], are excellent models to monitor circRNA expression and alterations during cancer development as this system faithfully recapitulates cancer evolution. Moreover, the impact of the potential qualitative/quantitative alterations of circRNAs on other factors, like RBPs and miRNAs, can also be experimentally monitored and provide critical answers on the pathways and processes affected by circRNA entities.

Similarly, 3D cellular systems have already been implemented in circRNA research, such as iPSC-derived brain organoids [95]. In those organoid cultures, 56% of the identified circRNAs overlapped with circRNAs of the postmortem brain [95]. Patient-derived organoids which are unique in their capacity to faithfully recapitulate the tissue of origin [96,97,98] have also been already employed to explore the role of circRNAs in certain types of cancer such as gastric cancer [99,100].

Other systems enabling the study of circRNAs are comprised by animal models in which loci encoding these molecules can be manipulated. Specifically, mice with a knockout of the *Cdr1as*circRNA locus that massively binds miR-7 and miR-671, displayed impaired sensorimotor gating, causing neuropsychiatric disorders from the inability of these animals to filter out unnecessary information [101]. Another study used shRNAs to target specific circRNA- back-splice junctions to specifically downregulate five highly expressed circRNAs in Droshophila [102]. Of note, downregulation of circ-Ctrip in this setting resulted in developmental lethality. These examples highlight the significance of such tools and provide important information on the functional significance and role of circRNAs.

## 8. Conclusions and Future Perspectives

CircRNAs represent an abundant and highly expressed group of regulatory RNAs, as depicted in several normal adult and fetal human tissues [103]. Moreover, they appear to have many functional implications, but most of them remain poorly characterized. A further issue that signifies their importance is their increasing prognostic/diagnostic role in various pathological conditions, including cancer. In the cancer field, DDRR-derived circRNAs are just starting to emerge as potentially valuable clinical tools, whose functionality and interplay with other players of the DDRR network is required to be thoroughly addressed. In this context, already available and newly established model systems like oncogene inducible cell systems, organoids or in vivo models may become useful platforms for validation experiments [94,96,97,98,99,100,101,102]. The thorough investigation of both the normal and cancer circRNA biology in various types of tissues is imperative as it may uncover unprecedented roles of the DDRR network and possibly new avenues for druggable therapeutic approaches, at a personalized level. 

## Supplementary Materials

The following are available online at https://www.mdpi.com/article/10.3390/cancers13215352/s1, Figure S1: Potential circRNAs derived from the NBS1 locus and potential “sponging” abilities, Figure S2: Potential circRNAs derived from the MRE11 locus and potential “sponging” abilities, Figure S3: Potential circRNAs derived from the RAD50 locus and potential “sponging” abilities, Figure S4: Potential circRNAs derived from the TP53BP1 locus and potential “sponging” abilities, Figure S5: Potential circRNAs derived from the CHK1 (CHEK1) locus and potential “sponging” abilities, Figure S6: Potential circRNAs derived from the CHK2 (CHEK2) locus and potential “sponging” abilities, Figure S7: Potential circRNAs derived from the ATM locus and potential “sponging” abilities, Figure S8: Potential circRNAs derived from the ATR locus and potential “sponging” abilities, Figure S9: Potential circRNAs derived from the TP53 locus and potential “sponging” abilities, Table S1: miRNAs and RBPs bound by DDRR-derived circDNAs-impact on cellular pathways.
