# Supplementary material for: The Role of Circular RNAs in DNA Damage Response and Repair
Source: Cancers (Basel). 2021 Oct 26;13(21):5352. doi: 10.3390/cancers13215352 (PMC8582540; doi:10.3390/cancers13215352)
Supplement: Supplementary file 1 [file cancers-13-05352-s001.zip › Supplementary figures.pdf]

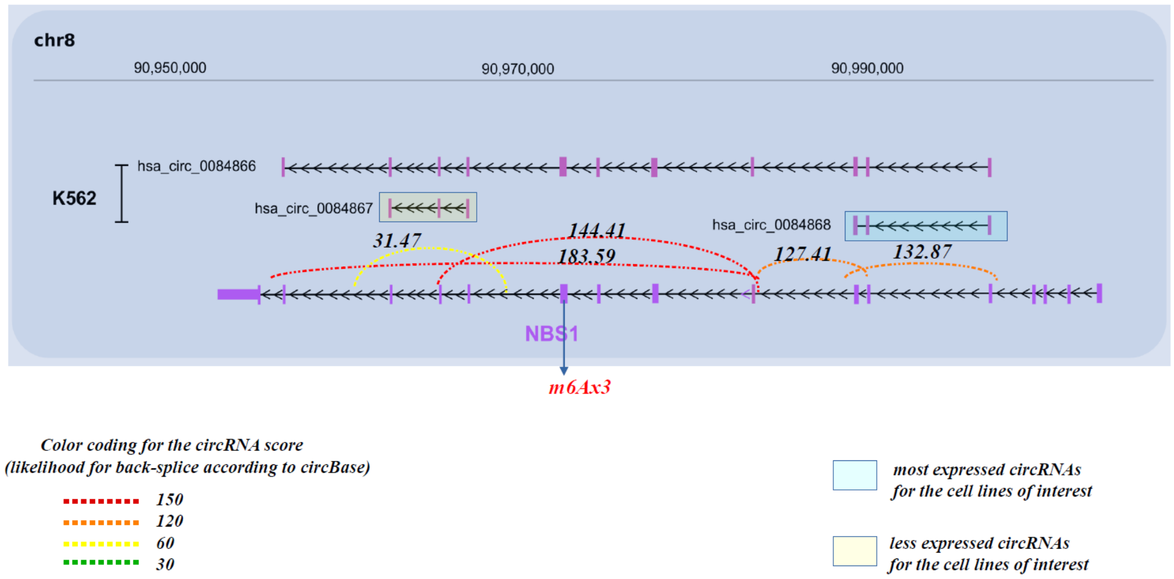

Figure S1. Potential circRNAs derived from the *NBS1* locus and potential "sponging" abilities. A. Predicted *NBS1*-derived circRNAs based on next generation sequencing (NGS) data obtained from depicted cancer cells lines as deposited at circBase (http://www.circbase.org/). With dashed arced lines we identified the most reliable back-splicing events. Color code ranging from red to orange to yellow, reflect a scale from very high to high scoring of the back-splicing events. Also the most expected versus less expected validation is shown. **B.** The most potent from the expected *NBS1*-derived circRNAs were examined for their binding capacity towards RBPs and miRNAs. For more details see Supplementary Table 1. **C.** Mutational profiles that overlap with the *NBS1* circular RNA transcripts [data from Circvar (http://soft.bioinfo-minzhao.org/circvar/), dbSNP (https://www.ncbi.nlm.nih.gov/snp/) and COSMIC (https://cancer.sanger.ac.uk/cosmic)] that can influence binding at putative miRNA and RBP interacting regions within the circular RNAs sequence and might alter its sponging capabilities and overall function.

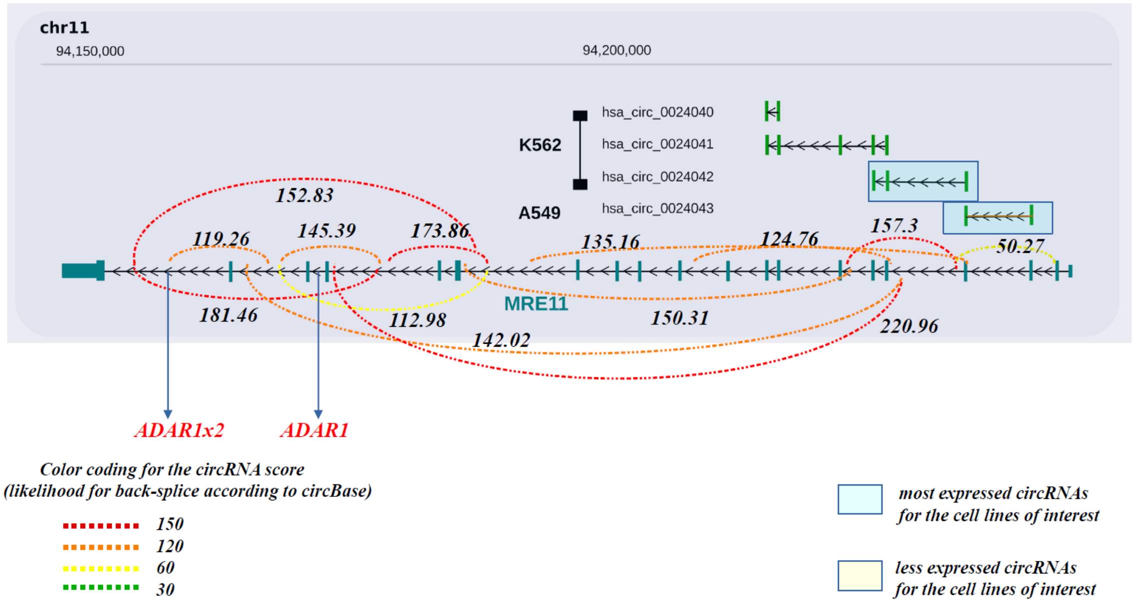

Figure S2. Potential circRNAs derived from the *MRE11* locus and potential "sponging" abilities. A. Predicted *MRE11*-derived circRNAs based on next generation sequencing (NGS) data obtained from depicted cancer cells lines as deposited at circBase (http://www.circbase.org/). With dashed arced lines we identified the most reliable

back-splicing events. Color code ranging from red to orange to yellow, reflect a scale from very high to high scoring of the back-splicing events. Also the most expected versus less expected validation is shown. **B.** The most potent from the expected *MRE11*-derived circRNAs were examined for their binding capacity towards RBPs and miRNAs. For more details see Supplementary Table 1. **C.** Mutational profiles that overlap with the *MRE11* circular RNA transcripts [data from Circvar (http://soft.bioinfo-minzhao.org/circvar/), dbSNP (https://www.ncbi.nlm.nih.gov/snp/) and COSMIC (https://cancer.sanger.ac.uk/cosmic)] that can influence binding at putative miRNA and RBP interacting regions within the circular RNAs sequence and might alter its sponging capabilities and overall function.

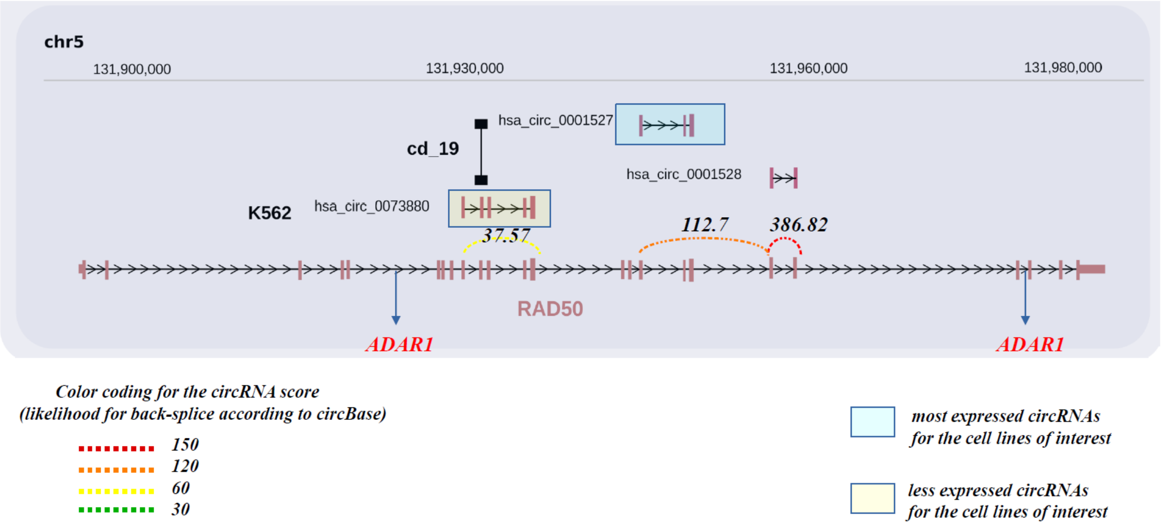

Figure S3. Potential circRNAs derived from the *RAD50* locus and potential "sponging" abilities. A. Predicted *RAD50*-derived circRNAs based on next generation sequencing (NGS) data obtained from depicted cancer cells lines as deposited at circBase (http://www.circbase.org/). With dashed arced lines we identified the most reliable back-splicing events. Color code ranging from red to orange to yellow, reflect a scale from very high to high scoring of the back-splicing events. Also the most expected versus less expected validation is shown. **B.** The most potent from the expected *RAD50*-derived circRNAs were examined for their binding capacity towards RBPs and miRNAs. For more details see Supplementary Table 1. **C.** Mutational profiles that overlap with the *RAD50* circular RNA transcripts [data from Circvar (http://soft.bioinfo-minzhao.org/circvar/), dbSNP (https://www.ncbi.nlm.nih.gov/snp/) and COSMIC (https://cancer.sanger.ac.uk/cosmic)] that can influence binding at putative miRNA and RBP interacting regions within the circular RNAs sequence and might alter its sponging capabilities and overall function.

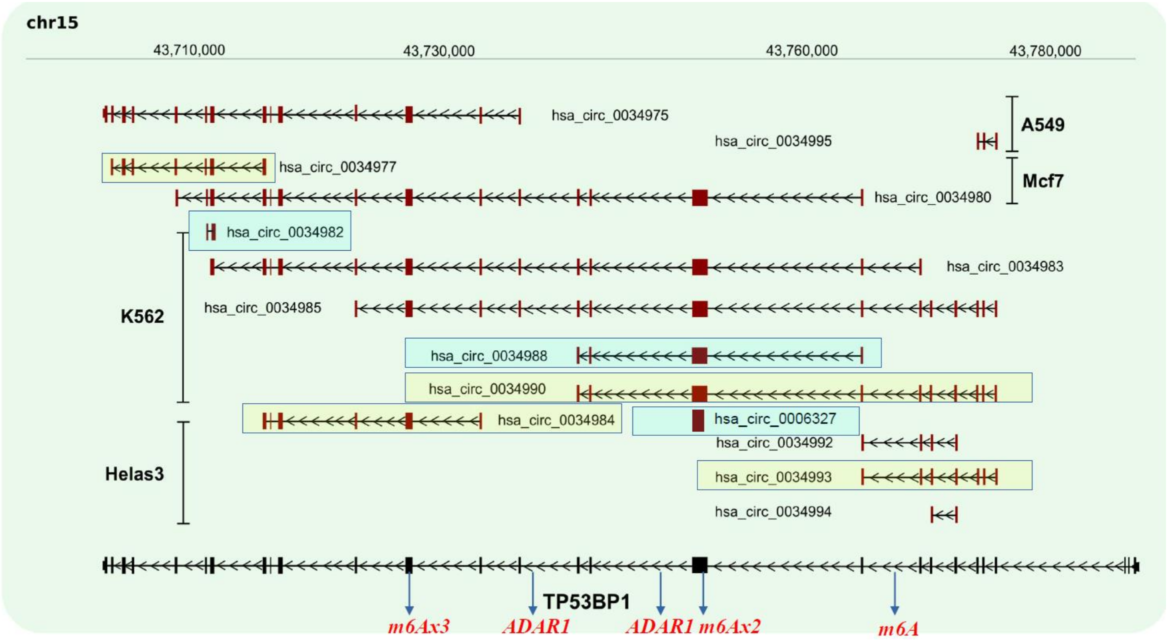

Figure S4. Potential circRNAs derived from the *TP53BP1* locus and potential "sponging" abilities. A. Predicted *TP53BP1*-derived circRNAs based on next generation sequencing (NGS) data obtained from depicted cancer cells lines as deposited at circBase (http://www.circbase.org/). With dashed arced lines we identified the most reliable back-splicing events. Color code ranging from red to orange to yellow, reflect a scale from very high to high scoring of the back-splicing events. Also the most expected versus less expected validation is shown. **B.** The most potent from the expected *TP53BP1*-derived circRNAs were examined for their binding capacity towards RBPs and miRNAs. For more details see Supplementary Table 1. **C.** Mutational profiles that overlap with the *TP53BP1* circular RNA transcripts [data from Circvar (http://soft.bioinfo-minzhao.org/circvar/), dbSNP (https://www.ncbi.nlm.nih.gov/snp/) and COSMIC (https://cancer.sanger.ac.uk/cosmic)] that can influence binding at putative miRNA and RBP interacting regions within the circular RNAs sequence and might alter its sponging capabilities and overall function.

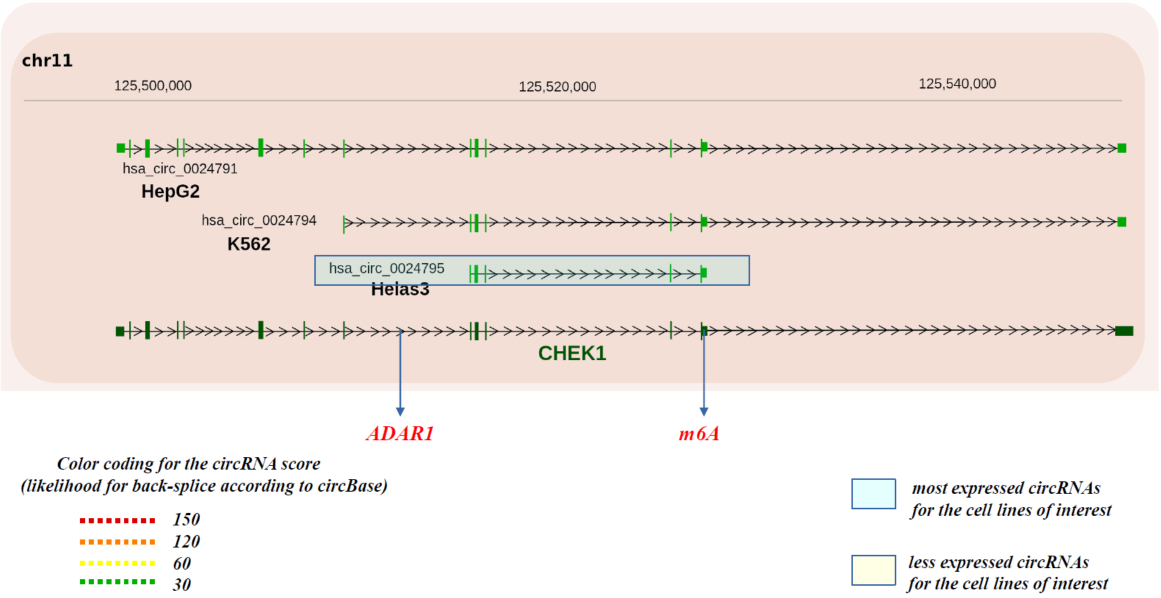

Figure S5. Potential circRNAs derived from the *CHK1 (CHEK1)* locus and potential "sponging" abilities. A. Predicted *CHK1*-derived circRNAs based on next generation sequencing (NGS) data obtained from depicted cancer cells lines as deposited at circBase (http://www.circbase.org/). With dashed arced lines we identified the most reliable back-splicing events. Color code ranging from red to orange to yellow, reflect a scale from very high to high scoring of the back-splicing events. Also the most expected versus less expected validation is shown. **B.** The most potent from the expected *CHK1*-derived circRNAs were examined for their binding capacity towards RBPs and miRNAs. For more details see Supplementary Table 1. **C.** Mutational profiles that overlap with the *CHK1* circular RNA transcripts [data from Circvar (http://soft.bioinfo-minzhao.org/circvar/), dbSNP (https://www.ncbi.nlm.nih.gov/snp/) and COSMIC (https://cancer.sanger.ac.uk/cosmic)] that can influence binding at putative miRNA and RBP interacting regions within the circular RNAs sequence and might alter its sponging capabilities and overall function.

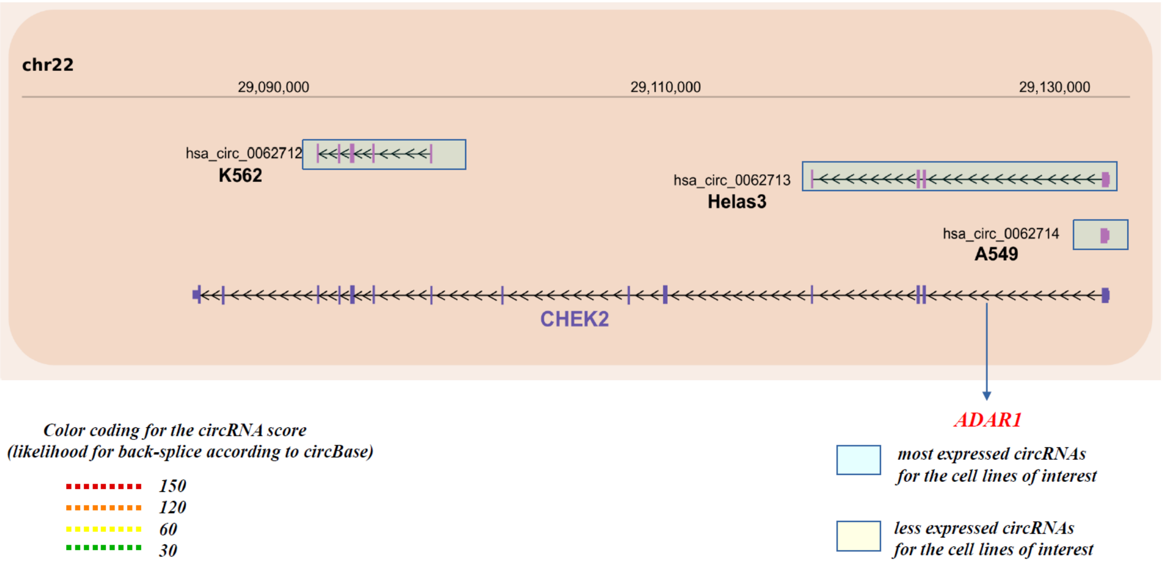

Figure S6. Potential circRNAs derived from the *CHK2 (CHEK2)* locus and potential "sponging" abilities. A. Predicted *CHK2*-derived circRNAs based on next generation sequencing (NGS) data obtained from depicted cancer cells lines as deposited at circBase (http://www.circbase.org/). With dashed arced lines we identified the most reliable back-splicing events. Color code ranging from red to orange to yellow, reflect a scale from very high to high scoring of the back-splicing events. Also the most expected versus less expected validation is shown. **B.** The most potent from the expected *CHK2*-derived circRNAs were examined for their binding capacity towards RBPs and miRNAs. For more details see Supplementary Table 1. **C.** Mutational profiles that overlap with the *CHK2* circular RNA transcripts [data from Circvar (http://soft.bioinfo-minzhao.org/circvar/), dbSNP (https://www.ncbi.nlm.nih.gov/snp/) and COSMIC (https://cancer.sanger.ac.uk/cosmic)] that can influence binding at putative miRNA and RBP interacting regions within the circular RNAs sequence and might alter its sponging capabilities and overall function.

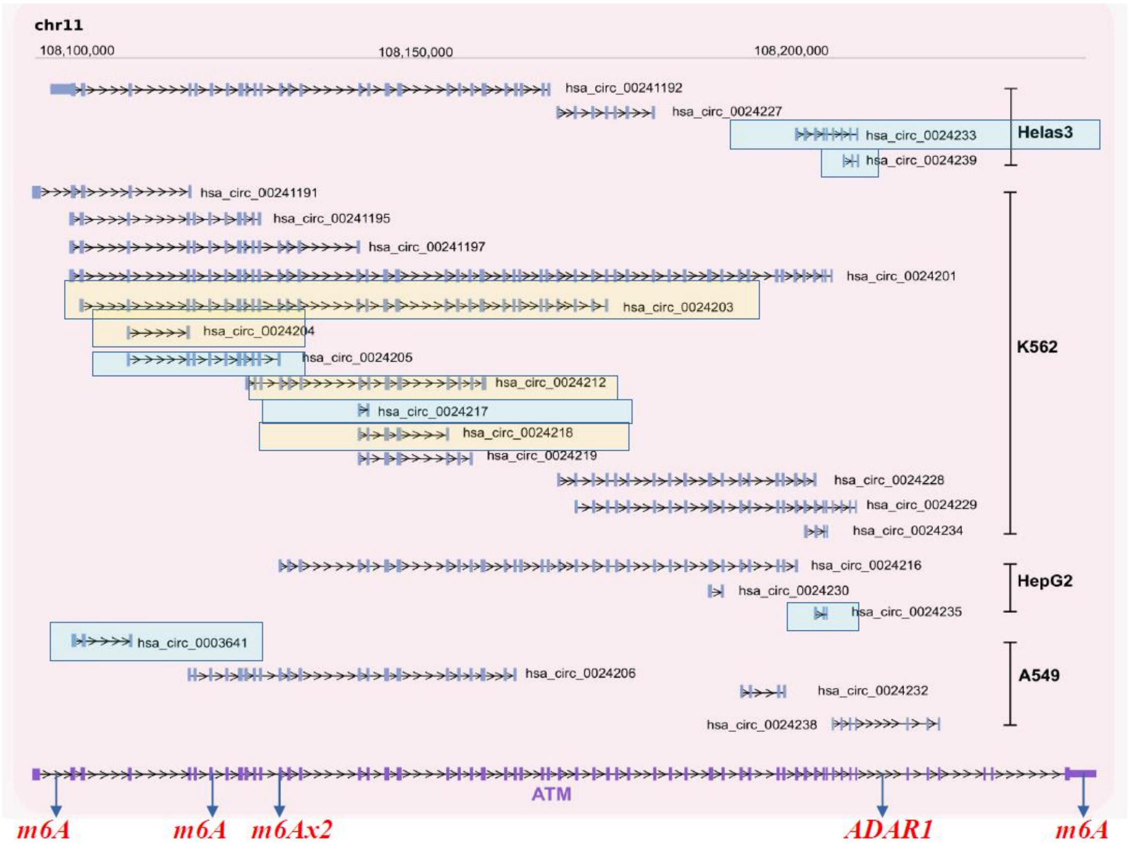

Figure S7. Potential circRNAs derived from the *ATM* locus and potential "sponging" abilities. A. Predicted *ATM*-derived circRNAs based on next generation sequencing (NGS) data obtained from depicted cancer cells lines as deposited at circBase (http://www.circbase.org/). With dashed arced lines we identified the most reliable back-splicing events. Color code ranging from red to orange to yellow, reflect a scale from very high to high scoring of the back-splicing events. Also the most expected versus less expected validation is shown. **B.** The most potent from the expected *ATM*-derived circRNAs were examined for their binding capacity towards RBPs and miRNAs. For more details see Supplementary Table 1. **C.** Mutational profiles that overlap with the *ATM* circular RNA transcripts [data from Circvar (http://soft.bioinfo-minzhao.org/circvar/), dbSNP (https://www.ncbi.nlm.nih.gov/snp/) and COSMIC (https://cancer.sanger.ac.uk/cosmic)] that can influence binding at putative miRNA and RBP interacting regions within the circular RNAs sequence and might alter its sponging capabilities and overall function.

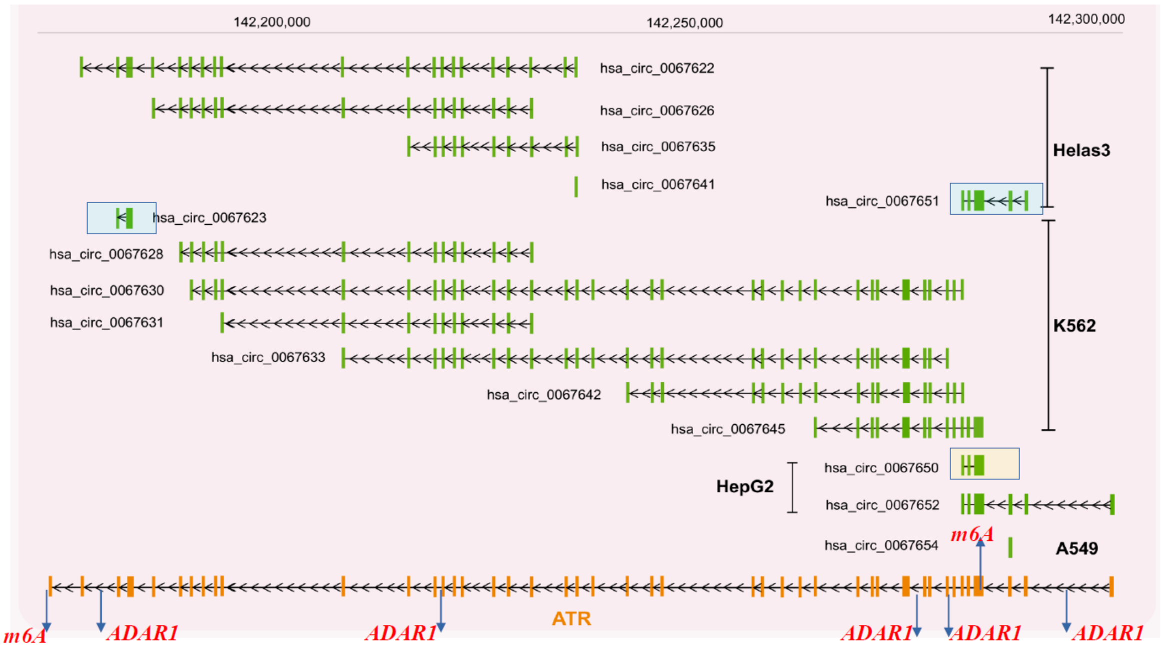

Figure S8. Potential circRNAs derived from the *ATR* locus and potential "sponging" abilities. A. Predicted *ATR*-derived circRNAs based on next generation sequencing (NGS) data obtained from depicted cancer cells lines as deposited at circBase (http://www.circbase.org/). With dashed arced lines we identified the most reliable back-splicing events. Color code ranging from red to orange to yellow, reflect a scale from very high to high scoring of the back-splicing events. Also the most expected versus less expected validation is shown. **B.** The most potent from the expected *ATR*-derived circRNAs were examined for their binding capacity towards RBPs and miRNAs. For more details see Supplementary Table 1. **C.** Mutational profiles that overlap with the *ATR* circular RNA transcripts [data from Circvar (http://soft.bioinfo-minzhao.org/circvar/), dbSNP (https://www.ncbi.nlm.nih.gov/snp/) and COSMIC (https://cancer.sanger.ac.uk/cosmic)] that can influence binding at putative miRNA and RBP interacting regions within the circular RNAs sequence and might alter its sponging capabilities and overall function.

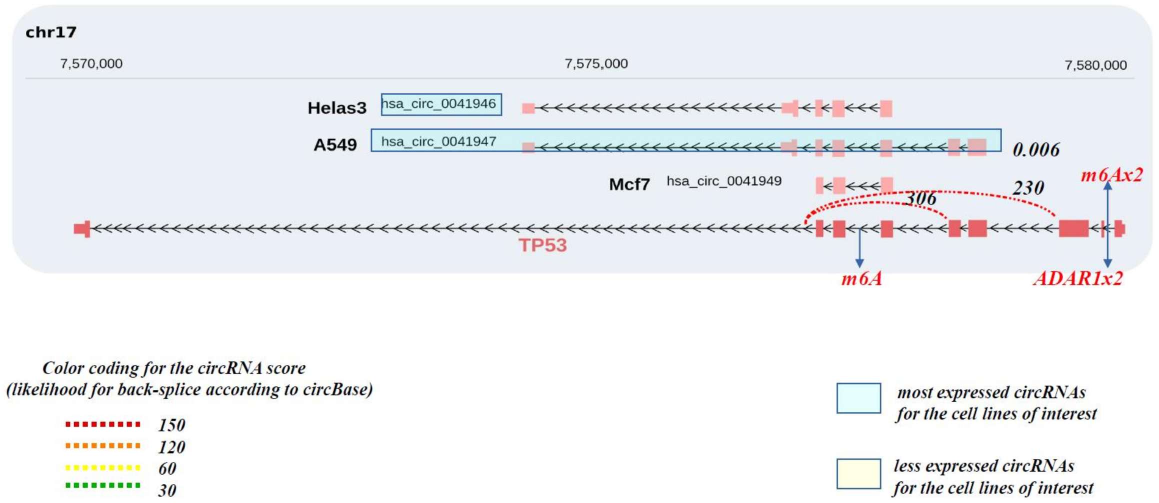

Figure S9. Potential circRNAs derived from the *TP53* locus and potential "sponging" abilities. A. Predicted *TP53*-derived circRNAs based on next generation sequencing (NGS) data obtained from depicted cancer cells lines as deposited at circBase (http://www.circbase.org/). With dashed arced lines we identified the most reliable back-splicing events. Color code ranging from red to orange to yellow, reflect a scale from very high to high scoring of the back-splicing events. Also the most expected versus less expected validation is shown. **B.** The most potent from the expected *TP53*-derived circRNAs were examined for their binding capacity towards RBPs and miRNAs. For more details see Supplementary Table 1. **C.** Mutational profiles that overlap with the *TP53* circular RNA transcripts [data from Circvar (http://soft.bioinfo-minzhao.org/circvar/), dbSNP (https://www.ncbi.nlm.nih.gov/snp/) and COSMIC (https://cancer.sanger.ac.uk/cosmic)] that can influence binding at putative miRNA and RBP interacting regions within the circular RNAs sequence and might alter its sponging capabilities and overall function.
